# Temporal scaling and computing time in neural circuits: Should we stop watching the clock and look for its gears?

**DOI:** 10.3389/fnbeh.2022.1022713

**Published:** 2022-12-08

**Authors:** Benjamin J. De Corte, Başak Akdoğan, Peter D. Balsam

**Affiliations:** ^1^Department of Psychology, Columbia University, New York, NY, United States; ^2^Division of Developmental Neuroscience, New York State Psychiatric Institute, New York, NY, United States; ^3^Department of Neuroscience and Behavior, Barnard College, New York, NY, United States

**Keywords:** time perception, drift-diffusion, population-clock, temporal scaling, temporal averaging, ramping activity, timing

## Abstract

Timing underlies a variety of functions, from walking to perceiving causality. Neural timing models typically fall into one of two categories—“ramping” and “population-clock” theories. According to ramping models, individual neurons track time by gradually increasing or decreasing their activity as an event approaches. To time different intervals, ramping neurons adjust their slopes, ramping steeply for short intervals and *vice versa*. In contrast, according to “population-clock” models, multiple neurons track time as a group, and each neuron can fire nonlinearly. As each neuron changes its rate at each point in time, a distinct pattern of activity emerges across the population. To time different intervals, the brain learns the population patterns that coincide with key events. Both model categories have empirical support. However, they often differ in plausibility when applied to certain behavioral effects. Specifically, behavioral data indicate that the timing system has a rich computational capacity, allowing observers to spontaneously compute novel intervals from previously learned ones. In population-clock theories, population patterns map to time arbitrarily, making it difficult to explain how different patterns can be computationally combined. Ramping models are viewed as more plausible, assuming upstream circuits can set the slope of ramping neurons according to a given computation. Critically, recent studies suggest that neurons with nonlinear firing profiles often scale to time different intervals—compressing for shorter intervals and stretching for longer ones. This “temporal scaling” effect has led to a hybrid-theory where, like a population-clock model, population patterns encode time, yet like a ramping neuron adjusting its slope, the speed of each neuron’s firing adapts to different intervals. Here, we argue that these “relative” population-clock models are as computationally plausible as ramping theories, viewing population-speed and ramp-slope adjustments as equivalent. Therefore, we view identifying these “speed-control” circuits as a key direction for evaluating how the timing system performs computations. Furthermore, temporal scaling highlights that a key distinction between different neural models is whether they propose an absolute or relative time-representation. However, we note that several behavioral studies suggest the brain processes both scales, cautioning against a dichotomy.

## Introduction

Time is a fundamental dimension of the world, and our nervous systems have likely been adapting to this fact since we began evolving. At a basic level, most motor programs, such as walking or riding a bicycle, require executing a series of well-timed and appropriately ordered actions. However, time also underlies many higher-order processes. For example, to comprehend causality, we must be able to recognize that causes *precede* their effects (Hume, 1739). When forming memories of personal experiences, we not only encode what happened and where, but also *when* different events occurred (Tulving, 1986). Relatedly, when we engage in decision-making, we often draw on prior experience to predict when future events will happen and adapt our behavior accordingly. Time governs virtually all facets of our daily lives, and in our view, the true challenge is finding a process that we engage in where time is entirely irrelevant. Therefore, uncovering how the brain represents time is a key topic in neuroscience.

Admittedly, “how does the brain track time?” is a remarkably broad question. However, there are ways to constrain the problem. For example, many sense modalities have dedicated organs (e.g., eyes for vision, cochlea for audition), yet there does not seem to be an analogous organ for processing time. In many ways, this aligns with common sense. We would be surprised if the system that allows us to effectively coordinate our footsteps in time were the same as the one that regulates our sleep-wake cycles over daily periods. Consistent with this, we now know that circadian timing is largely regulated by gene-expression rhythms in the suprachiasmatic nucleus (Drucker-Colín et al., 1984; Silver et al., 1996). In contrast, millisecond-timing, being important for motor coordination/calibration, relies on neural activity in regions such as primary sensory/motor areas (Long et al., 2016), the cerebellum (Garcia and Mauk, 1998), and central pattern generators in the spinal cord (Kudo and Yamada, 1987). Therefore, when asking how the brain tracks time, we can hone the question by choosing a timescale of interest (for further discussion see Buhusi and Meck, 2005; Paton and Buonomano, 2018).

In the present article, we will focus on “interval timing,” which generally falls in the seconds-to-hours range. Interval timing underlies a variety of processes, such as associative learning (Gallistel and Gibbon, 2000; Balsam and Gallistel, 2009), causal-inference (Roberts and Holder, 1984; Fereday et al., 2019), and decision-making (Balci et al., 2009a; Gür et al., 2018). It recruits a complex network, including (but not limited to) several association cortices (Leon and Shadlen, 2003; Merchant et al., 2013; Bakhurin et al., 2017; Buhusi et al., 2018), thalamic nuclei (Komura et al., 2001; Wang et al., 2018; Lusk et al., 2020), the basal ganglia (Gouvêa et al., 2015; Mello et al., 2015), and midbrain dopamine centers (Meck, 2006; Soares et al., 2016; Howard et al., 2017). Despite extensive investigation, little consensus has emerged over how these areas encode time or interact to support temporal processing. However, recent experiments have consistently pointed to a general principle by which the brain might accomplish interval timing—referred to as “temporal scaling.” Part of this manuscript will serve as a concise review of these data. However, we will give more emphasis to how these findings: (1) recontextualize past theoretical disputes in the field; (2) reframe the distinctions we draw between different timing models; and (3) provide clear future directions for studying the neural mechanisms of timing.

## The General Schema of Most Interval Timing Models

Theorists have developed a variety of neural models of timing (Grossberg and Schmajuk, 1989; Matell and Meck, 2004; Buonomano and Maass, 2009; Simen et al., 2011; Wang et al., 2018). The specifics of one theory often differ substantially from the others. However, at a general level, most models segregate the timing system into the same core set of subprocesses. To frame a more detailed theoretical discussion below, we will first overview these similarities.

**Figure 1:**
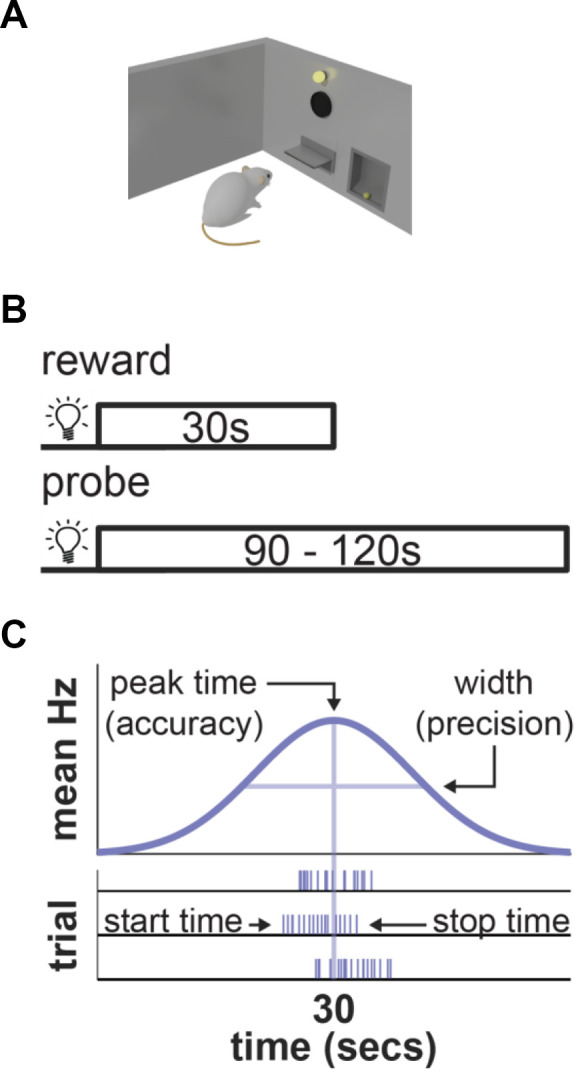
Peak-interval procedure. **(A)** Illustration of task equipment. **(B)** Task-design diagram. **(C)** Illustration of ideal behavior during probe trials with respect to mean response rates (top) and single-trials (bottom).

To illustrate with a concrete example, consider how one would develop a model that explains behavior during the “peak-interval” procedure—a common interval timing task (Roberts, 1981). This task can be used with a variety of species (mice: Balci et al., 2009b; rats: Roberts, 1981; goldfish: Drew et al., 2005; humans: Rakitin et al., 1998). However, as much of our discussion will center on data from rats, we will illustrate how the task applies to this species specifically (Figure 1). During this task, experimenters place rats in an operant chamber, containing a response-manipulandum (e.g., lever). Occasionally, a cue (e.g., light) turns on that signals that reward can be earned for responding after a specific duration elapses (e.g., 30 s). During some trials, the first response a rat makes after this interval has passed causes the cue to turn off and the reward to be delivered. However, experimenters also include a subset of “probe” trials during which the cue remains on for much longer than normal (e.g., 90–120 s) and no reward is provided (Figure 1B).

During a probe trial, a rat must know when to begin responding in anticipation of a reward and when to stop responding once the target-interval has passed. Consistent with this, a trained rat will typically emit a discrete cluster of responses that fall around the target interval–usually beginning before and ending after it elapses (Figure 1C; Gibbon and Church, 1990). When the cluster starts and stops (simply referred to as the start- and stop-times) will vary slightly from one trial to the next. Consequently, when averaged across trials, response rates plotted across time will resemble a normal distribution, with a peak (i.e., “peak time”) centered over the target duration. How close the peak-, start-, and stop-times fall to the true target interval indicate how accurately a rat is performing. Furthermore, the variability of start- and stop-times—as well as the width of the mean response distribution—reflect how precisely a rat is timing.

To construct a model that explains performance during this task, we would want to capture three general processes that the brain must implement to guide performance. First, it must obviously contain a time-keeping mechanism that allows it to track time as it passes, particularly the moment of reward. Second, to use the time of reward during future trials, the brain must be able to represent it in memory. Finally, the brain must contain a decision-making mechanism that allows the two above processes to guide behavior, executing responses near the remembered interval. As highlighted below, virtually all neural models of timing (and also most behavioral ones; e.g., Gibbon et al., 1984; Killeen and Fetterman, 1988; Church and Broadbent, 1990; Machado, 1997) address these three core functions, yet they can differ markedly in their proposals over how each is implemented.

## Classes of Timing Models

For brevity, we will avoid giving an exhaustive overview of existing neural theories of timing. Instead, we will describe the two most dominant model categories—ramping and population-clock theories—and provide one specific example of each.

### Ramping models

**Figure 2:**
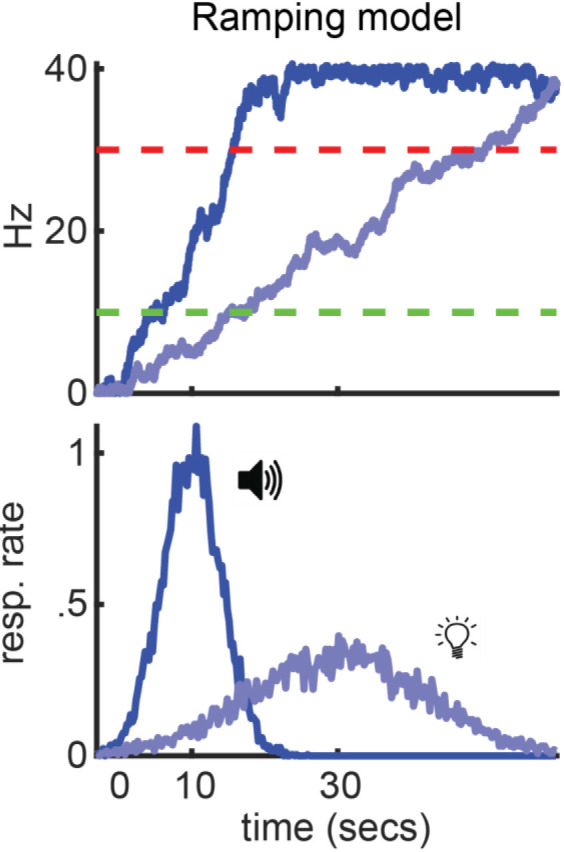
Ramping model. Summary of how a ramping model would apply to a peak procedure where a tone and light signal reward after 10 and 30 s, respectively. Top: Simulated ramps for each cue (blue) and start/stop thresholds (green and red, respectively). Bottom: Simulated, idyllic behavior.

According to ramping models, time-keeping is accomplished via individual neurons that gradually increase or decrease their activity across time–aptly referred to as “ramping” neurons (Figure 2; Durstewitz, 2003; Reutimann et al., 2004; Gavornik et al., 2009; Simen et al., 2011). Subjects execute decisions when these neurons reach particular firing rates, referred to as “decision thresholds.” Importantly, to time these decisions effectively, a ramping neuron will adjust its slope to reach these thresholds near the target interval, ramping faster for shorter intervals and slower for longer ones. The slope corresponds to the temporal memory associated with the cue, as it varies systematically with its trained interval. These proposals are well supported by empirical data. For example, researchers have repeatedly observed ramping neurons while subjects perform timing tasks (Niki and Watanabe, 1979; Leon and Shadlen, 2003; Matell et al., 2011; Xu et al., 2014; Jazayeri and Shadlen, 2015). Furthermore, ramping neurons adapt their slopes to different target intervals, and time-based decisions often coincide with when they reach particular firing rates (Komura et al., 2001; Leon and Shadlen, 2003; Jazayeri and Shadlen, 2015; Murakami et al., 2017).

In Figure 2, we show how a ramping model would apply to the peak-interval procedure specifically (Luzardo et al., 2017a). To highlight the importance of slope adjustments for timing different durations, we use an example where two cues—presented during separate trials—signal reward after different target intervals (tone=10s / light=30s). Regardless of what cue is presented, the ramping neuron begins ascending at the start of a trial. Furthermore, responding will begin when it reaches the start-threshold and end when it reaches the stop-threshold (green and red lines, respectively). However, to reach these thresholds at the appropriate time for each cue, the ramping neuron takes a steeper slope when the short-cue is presented, relative to the long-cue.

**Figure 3:**
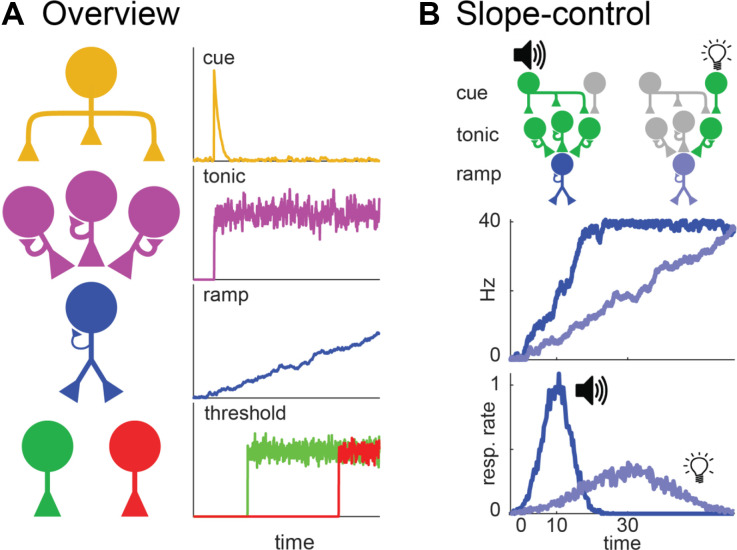
Simen et al. (2011) ramping model. **(A)** Circuit architecture for cue, tonic, ramping, and threshold units, including purported firing profiles of each to the right according to the variant developed by De Corte (2021). **(B)** Slope control within the model during a task where a tone and light signal a 10 and 30 s interval, respectively. Top: Circuit-architecture for a dual-cue task as proposed by De Corte (2021). Each cue elicits a response from a different cue unit. Each unit activates the number of tonic units needed to achieve the ramp-slope for its respective interval. The middle and bottom are analogous to Figure 2; showing simulated ramping and behavior.

Simen et al. (2011) proposed what is arguably the leading ramping theory of timing. Figure 3 summarizes how it applies to the peak-interval procedure (for further details on this particular adaptation see De Corte, 2021). Specifically, Figure 3A overviews the model’s architecture, which resembles a neural network with four layers that interact hierarchically to generate ramping activity and timed responding. Units in the top-layer function much like simple sensory-neurons. When a cue is presented at the start of a trial, units in this layer that code for the stimulus will fire transiently. These “cue-units” send excitation to the second layer, containing what we will call “tonic units.” Tonic units possess strong self-excitation, causing each output they produce to also act as a potent, depolarizing input. Consequently, when initially excited by cue-units, they will quickly begin firing maximally throughout the trial (i.e., tonically), due to recurrent-excitation alone. This steady excitation outputs to the third layer, composed of ramping units. Ramping units also possess recurrent-excitation. However, unlike tonic units, it is too weak to cause them to ascend to their maximum firing rates, yet still strong enough to prevent firing-rate decay. This tuned recurrent-excitation effectively allows ramping units to summate input from the tonic layer from one moment to the next. As a result, ramping units will assume a linear profile, with the slope depending on the net excitation they receive from the tonic layer, per unit time. Finally, the ramping layer outputs to “threshold” units that only become active when the ramp reaches specific firing rates. These units control the circuit’s behavioral output. Specifically, start-threshold units activate at a moderate input-level from the ramping layer, initiating responses. Then, once the ramp ascends to an even higher activity level, stop-threshold units activate, terminating responding.

As noted above, the key parameter in any ramping model is how to adjust the slope of the ramping neuron to time different intervals. In Simen et al.’s (2011) model, the slope is determined by the net excitation the ramping layer receives from the tonic layer (i.e., higher excitation = steeper slope). Therefore, to adjust the slope, one simply adjusts the number of tonic units that the cue-layer excites at the start of a trial (De Corte, 2021). Figure 3B clarifies this more concretely, keeping with the example where a tone and light signal a 10 and 30 s target interval, respectively. When the tone is presented, one cue-unit activates all three of the diagrammed tonic-units. With high-excitation from the tonic layer, the ramp ascends at a steep slope, yielding responses at 10 s. In contrast, when the light is presented, a different cue-unit fires that only activates one tonic unit. With less net input from the tonic layer, the ramp takes a shallower slope, yielding responses at 30 s.

### Population-clock models

Population-clock theories of timing primarily differ from ramping models with respect to how time-keeping is accomplished. As described above, ramping models propose that individual ramping neurons track time, and their rates at any moment covary linearly with how much time has passed. In contrast, according to population-clock models, a group of neurons tracks time collectively, and the current rate of a given neuron does not have to map systematically to elapsed time (Ahrens and Sahani, 2007; Karmarkar and Buonomano, 2007; Laje and Buonomano, 2013). In fact, the firing profile of each individual neuron can effectively be random. The key point is that, so long as each neuron repeats its temporal firing-profile across trials, a distinct pattern of activity will emerge across the population at each moment, providing a readout of elapsed time.

**Figure 4:**
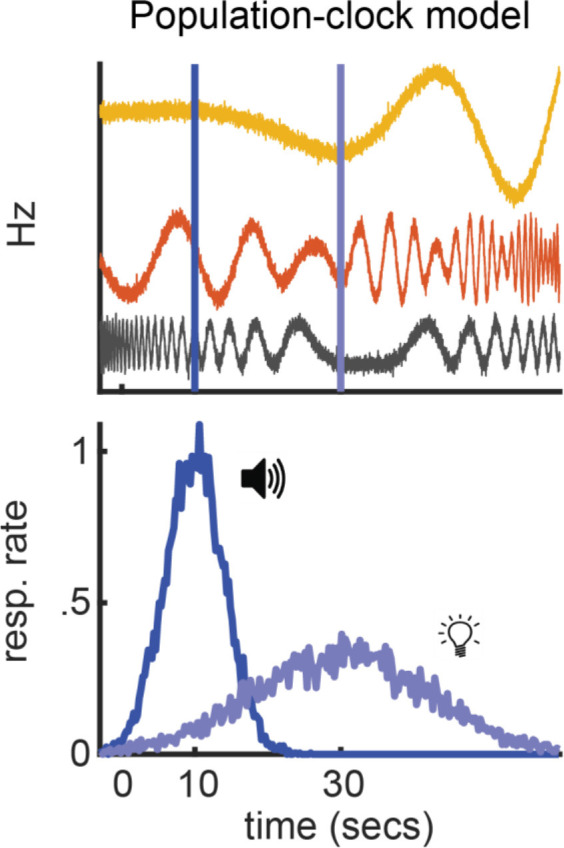
Population-clock model. Simple population-clock model during a peak-procedure where a tone and light signal reward after 10 and 30 s, respectively. Top: Nonlinear population activity from three neurons. Bottom: Behavior coincides with each cue’s pattern.

We find it easiest to convey this point with a simple example, as illustrated in Figure 4 for the peak-interval procedure. At the start of a trial, three simulated neurons begin to fire. We have deliberately programmed each neuron’s rate to fluctuate erratically across time. By definition, there is no direct relationship between an individual neuron’s current firing rate and elapsed time. However, if each neuron’s firing profile repeats across trials, a unique pattern of activity will emerge across the population for each moment within a trial. Therefore, if the brain can learn the patterns that coincide with critical task events (i.e., the times of reward for each cue), it can guide behavior in time. Importantly, under this regime, the brain can time any target interval by tuning to its corresponding activity pattern. This is the premise of virtually all population-clock models. Neurons do not encode time individually. Rather, time is represented by an evolving pattern of activity across a group of neurons, which functions as a population clock.

While individual neurons can take any shape to contribute to a population-clock, some models propose constraints on how time-keeping neurons fire, based on *in vivo* data and/or biophysically realistic assumptions. For example, some theories align closely with the simple model above, proposing that neurons contribute to timing via highly dynamic profiles (Buonomano and Merzenich, 1995; Ahrens and Sahani, 2007). However, others propose that neurons encode time via gaussian-like profiles specifically, with different neurons peaking at distinct moments within a timed-interval (Shankar and Howard, 2012; Zhou et al., 2020). Consistent with this, neural ensembles often display sequential activity during timing tasks that collectively “tiles” each moment between the start and end of a target-interval (MacDonald et al., 2011; Mello et al., 2015; Bakhurin et al., 2017; Tiganj et al., 2017; Shimbo et al., 2021). These are often referred to as “time-cells,” particularly when observed in the hippocampus (Eichenbaum, 2014), and while individual neurons do keep time to some degree within these models, a full population is still required to encode the entire interval. Yet, other models propose that individual neurons fire in an oscillatory manner, with periodicities that vary across neurons in the population (Miall, 1989; Matell and Meck, 2004). These models are inspired by the known mechanisms of circadian-timing—gene-expression oscillations (Silver et al., 1996)—and behavioral theories of interval timing (Church and Broadbent, 1990). Admittedly, there is some disagreement over whether these models should be grouped together as population-clock theories (for excellent discussion see Mauk and Buonomano, 2004; Paton and Buonomano, 2018). Fortunately, while this is important to note, we can avoid these nuances for the present article. All of these models share the common premise that time is encoded by patterns of activity across groups of neurons rather than by individual neurons—the broad definition of a population-clock theory we rely on here. As we will see shortly, all the following claims will apply across them.

**Figure 5:**
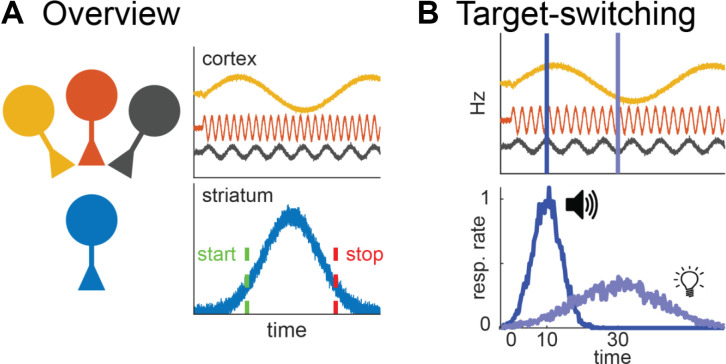
Striatal beat frequency model. **(A)** Model overview showing cortical oscillators projecting onto a striatal unit. Firing profiles for each layer to the right. **(B)** Timing different intervals. Striatal neurons tune to the cortical pattern appropriate for a given cue’s interval.

The Striatal-Beat Frequency (SBF) model is an example of a population-clock model that has been applied extensively to the peak-interval procedure (Matell and Meck, 2004; Oprisan and Buhusi, 2014). Consistent with the oscillation-based models mentioned above, SBF proposes that, when a trial starts, frontal cortical neurons implement time-keeping by beginning to fire in an oscillatory manner (Figure 5A, top). Different neurons fire at different periodicities, yielding a unique pattern of activity across the population at each moment. These cortical oscillators project to individual striatal neurons (Figure 5A, bottom). Striatal neurons implement both the memory and decision processes, learning the cortical pattern that coincides with the target interval and, once established, triggering responses when it emerges. Specifically, early in training, reward-delivery elicits a phasic burst of nigrostriatal dopamine (e.g., Amo et al., 2022). Dopamine strengthens active corticostriatal synapses and weakens inactive ones. As such, striatal neurons become more responsive to the reward-related activity pattern. Once this plasticity is established, midbrain dopamine neurons transition to firing at cue-onset. This purportedly resets striatal neurons to their resting potentials, sharpening their sensitivity to the critical cortical pattern. As the pattern emerges and dissipates, striatal neurons fire accordingly and, in turn, generate behavioral output. To time a different interval, striatal neurons would simply re-tune to its respective activity pattern (Figure 5B).

## Evidence for Population-Clock and Ramping Models: The Importance of Behavioral Work

Having covered the basics of population-clock and ramping models in the prior section, we now turn to how they have been empirically evaluated in the past. A common (and perhaps the most obvious) approach is to monitor neural activity *in vivo* during a timing task and assess how well the data match with either model-class. However, most neurophysiological studies have found mixed support for both types. As noted above, experimenters have repeatedly documented ramping neurons during timing tasks (Jazayeri and Shadlen, 2015; Li et al., 2016). Nonetheless, consistent with population-clock models, investigators typically find neurons with nonlinear firing profiles as well, ranging from sequential gaussian-like activity to more complex dynamics (Matell et al., 2011; Gouvêa et al., 2015; Mello et al., 2015; Zhou et al., 2020). As such, the neurophysiological data alone have been inconclusive.

In an ideal world, we would simply inhibit ramping or nonlinear neurons selectively to evaluate the roles they might play in timing. However, a key obstacle is that ramping and nonlinear neurons typically co-exist within the same population (Matell et al., 2011; Zhou et al., 2020). Consequently, without substantial advances in methods for manipulating specific neurons in the same population *in vivo* (e.g., holographic optogenetics Pégard et al., 2017), causally testing whether one neuron-type plays a preferential role in timing will be effectively impossible. Therefore, investigators have had to use alternative approaches to assess the plausibility of ramping and population-clock models.

Behavioral studies have been helpful in this regard. Ultimately, all neural models must explain key behavioral effects that occur during timing tasks. As a classic example, the standard deviation of time-estimates often varies linearly with the target interval–referred to as the “scalar property” of interval timing (Gibbon, 1977). To illustrate with the peak-procedure, if a cue’s target-interval suddenly doubles from 10 to 20 s, both the mean and standard deviation of start and stop times will double, in addition to the peak-time/spread of mean responding. Explaining how this locked accuracy-precision relationship emerges from neural circuits is often non-trivial. Therefore, the scalar property is a standard starting-constraint on any neural model of timing. Critically, other effects speak more directly to the distinction between ramping and population-clock models.

**Figure 6:**
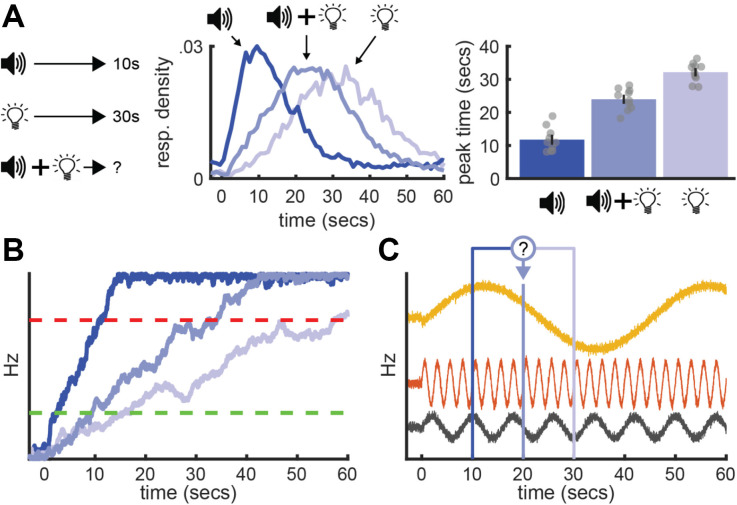
Temporal averaging and neural models of timing. **(A)** Temporal averaging behavior. Left: Example task-schematic for a temporal averaging experiment. Middle: Mean response rates across time during each trial type. Right: Peak-times for each trial type. Data adapted from De Corte (2017). **(B)** Typical account of temporal averaging under a ramping model, wherein a ramping neuron takes an intermediate slope during compound trials. **(C)** Application of a population-clock model (here, SBF) to temporal averaging. The question-mark emphasizes the ambiguity over how two population patterns could be integrated to yield a pattern between the two target times.

A notable example is a behavioral effect referred to as “temporal averaging” (Swanton et al., 2009). Originally, temporal averaging experiments were developed to test how the timing system handles conflicting time-information regarding when an upcoming event will occur. Typically, experimenters begin by training rats on a standard peak-interval procedure that incorporates two cues—each associated with a distinct target interval (e.g., tone=10s / light=30s). After training each cue individually, experimenters introduce trials where both cues are presented simultaneously as a “compound” stimulus. The key question is how rats will spontaneously react to suddenly being presented with two cues that predict reward after different intervals (i.e., conflicting time-information). Therefore, experimenters never train rats to respond at a specific time during compound trials by delivering the reward. Remarkably, during compound trials, rats often respond in between the two-cue’s intervals in a unimodal, scalar manner (Swanton et al., 2009; Swanton and Matell, 2011; Kurti et al., 2013; Matell and Kurti, 2014; Delamater and Nicolas, 2015; De Corte and Matell, 2016a; Matell et al., 2016; Shapiro et al., 2018). In other words, when conflicting temporal cues are presented, the timing system appears to integrate the information each signal provides into an average interval, timing this average in an otherwise normal way. Figure 6A shows example data from De Corte (2017) who, much like the example we have been using thus far, trained rats to associate a tone and light with a 10 and 30 s duration, respectively. While temporal averaging has been studied most extensively in rats (for review see De Corte and Matell, 2016b), it was originally documented in pigeons (Cheng and Roberts, 1991). Furthermore, humans show this effect (Zeng and Chen, 2019), in addition to various forms of non-temporal averaging behavior during other tasks involving conflicting information (for review see Fetsch et al., 2013).

While interesting in its own right, many have noted the implications of temporal averaging for neural models of timing (Matell and Henning, 2013; Matell, 2014; Matell and De Corte, 2016; Raphan et al., 2019). To illustrate, consider how a ramping model might account for this effect, as summarized in Figure 6B. When a cue is presented individually, ramping neurons would set their slopes according to their respective interval, ramping faster for the shorter cue than the long. Accordingly, during compound trials, one might expect ramping neurons to take an intermediate slope, thereby reaching the decision thresholds at an intermediate-time (De Corte and Matell, 2016b; Luzardo et al., 2017b). While straightforward, a deterministic explanation for why a ramping neuron would adopt an intermediate slope during compound trials has been more elusive. We recently developed solutions under Simen et al.’s (2011) model (De Corte, 2021). However, for brevity, we will avoid detailing these possibilities here. For present purposes, the primary point is that, on its face, temporal averaging does not appear to be fundamentally incompatible with ramping models.

In contrast, explanations for temporal averaging based on population-clock theories have been more elusive (Matell and Henning, 2013; Matell, 2014). Take SBF as an example (Figure 6C). When a single cue is present, striatal neurons would tune to the cortical oscillatory pattern coinciding with its target interval. Following from this, during compound trials, striatal neurons would presumably tune to a pattern that falls in etween the two cues’ intervals. However, there are several problems with this explanation. For example, as discussed above, SBF proposes that striatal neurons only become responsive to a cortical pattern via a reward-dependent learning process. As reward is never delivered during compound trials, this explanation is not viable.

To resolve this, some have considered whether a more “top-down” mechanism could be added to the model that drives striatal neurons to the pattern associated with the average interval (Matell and Henning, 2013). However, this line of inquiry has led to what might be a more fundamental problem with the model. Specifically, how could the pattern associated with an average interval be computed from the patterns associated with each cue’s duration? After all, time is not represented in a “quantitative” way within SBF. Rather, in statistical terms, the cortical patterns that occur at different moments can be thought of as nominal variables. Each pattern can be swapped for another, and as long as they unfold in a consistent manner across trials, the method for timing would remain the same. Therefore, “averaging” two cortical patterns would be as meaningful as averaging two telephone numbers. As such, how information can be integrated across cues that lead to temporal averaging—or responding at any novel duration that is systematic across subjects—is unclear.

Critically, because a defining feature of all traditional population-clock theories is a nominal mapping of population activity to time, they will face the same challenge when applied to temporal averaging. Moreover, temporal averaging is not the only behavioral effect where this “computational intractability” becomes a problem. Rather, it will emerge whenever subjects appear to compute higher-order information across different cues/intervals. The behavioral literature provides several added examples worth noting.

One that relates closely to temporal averaging is an effect referred to as “Vierordt’s law.” This effect has been documented most extensively in humans and occurs during tasks where subjects time different intervals that vary from one trial to the next. Under these conditions, a subject’s estimate for a given interval will regress toward the mean of all intervals they have previously learned, overshooting shorter intervals and undershooting longer ones (Lejeune and Wearden, 2009; Jazayeri and Shadlen, 2010). Parkinson’s patients show a pronounced form of Vierordt’s law, prompting a unique label called the “migration effect” (Malapani et al., 1998, 2002). Bayesian models account for this effect by assuming subjects offset potential errors in time-estimation during a trial by integrating their current estimate with prior knowledge regarding intervals that are typically presented in a given context (Jazayeri and Shadlen, 2010; Shi et al., 2013). The more a given estimate deviates from all previously learned intervals (i.e., the more “unusual” it is for a given task), the more it will be corrected based on prior knowledge. Consequently, all estimates will naturally regress toward the mean of the prior distribution. Again, population-clock theories face difficulty accounting for this integration process. However, within ramping models, one would assume that the slope of ramping neurons is biased toward that associated with the mean interval, and again, mechanistic explanations regarding why this would occur are beginning to emerge (De Corte, 2021).

Averaging-related effects are not the only instances where information is flexibly integrated during timing tasks (for excellent reviews see: Molet and Miller, 2014; Gür et al., 2018). For example, in a recent series of experiments, we showed that rats expect the intervals associated with distinct cues will covary with one another (De Corte et al., 2018, 2022). In other words, when one cue’s interval changes, they expect other cues’ intervals to have changed in the same direction and can even flexibly update this covariance expectation based on task evidence. In mathematical terms, computing expected covariance (or a rough approximation to it) requires a unique set of operations relative to averaging numbers, posing a distinct challenge for neural models of timing. Furthermore, subjects also appear to be able to integrate temporal and non-temporal information. For instance, when timing two intervals that require a response at different locations, mice will flexibly integrate time-information with reward-probability information at each location in a roughly optimal manner (Balci et al., 2009a).

Again, while biophysically deterministic explanations for these effects are forthcoming in any timing model, population-clock network models face a particularly difficult challenge due to their nominal time-representation.

## Temporal Scaling: Implications for Computation in Population-Clock Models

In the above section, we argue that: (1) behavioral experiments have repeatedly demonstrated that the timing system has a rich computational capacity, and (2) implementing these computations using population-clock models is particularly challenging. To be clear, we do not claim that these “higher-order” behavioral effects are categorically impossible within traditional population-clock theories. For example, much like the arbitrary association between time and population patterns they propose, computers use arbitrary codes to represent numeric values–composed of unique bit-sequences. However, to carry out mathematical operations on these codes, computers must be equipped with specific circuits that are tailored for a given mathematical function and built around the agreed-upon mapping between a given sequence and its corresponding quantity. In principle, population-clock theories could be equipped with neural-parallels to these circuits, along with a sequence-quantity convention. After all, perceptron-circuits allow for Boolean logic gates—the fundamental building block of any computer or computational circuit—to be implemented *in silico* (McCulloch and Pitts, 1943). Nonetheless, this would entail a substantial revision to current population-clock theories. Furthermore, once modified, whether they would continue to capture basic patterns of timing-behavior (e.g., the scalar property) and known-patterns of neural activity during timing tasks would have to be re-evaluated. Critically, recent data point to a simpler solution, which we turn to next.

These data relate to a less obvious distinction between population-clock and ramping models that we have not emphasized thus far. To preface, note that the advantage of ramping models when applied to the above behavioral effects does not relate to the linear-shape of ramping profiles, *per se*. Rather, it is the assumption that upstream circuits can flexibly set the slope of ramping neurons as needed for a given computation. As an example, if these circuits can compress or stretch a ramping profile to time a short or long interval, we assume they can do the same to time an intermediate interval during a temporal averaging experiment. The key point here is that slope adjustments produce a *relative*-mapping of neural activity to time, as a ramping neuron’s rate at any moment will reflect the *fraction* of the target-interval that has elapsed.

In contrast, traditional population-clock models propose an *absolute*-mapping of neural activity to time. Regardless of whether the target-interval is 10 or 30 s, the neural pattern at 5 s will be the same. As it is unclear how these population patterns can be combined in a computationally useful way, this absolute-mapping produces the challenges discussed above.

**Figure 7:**
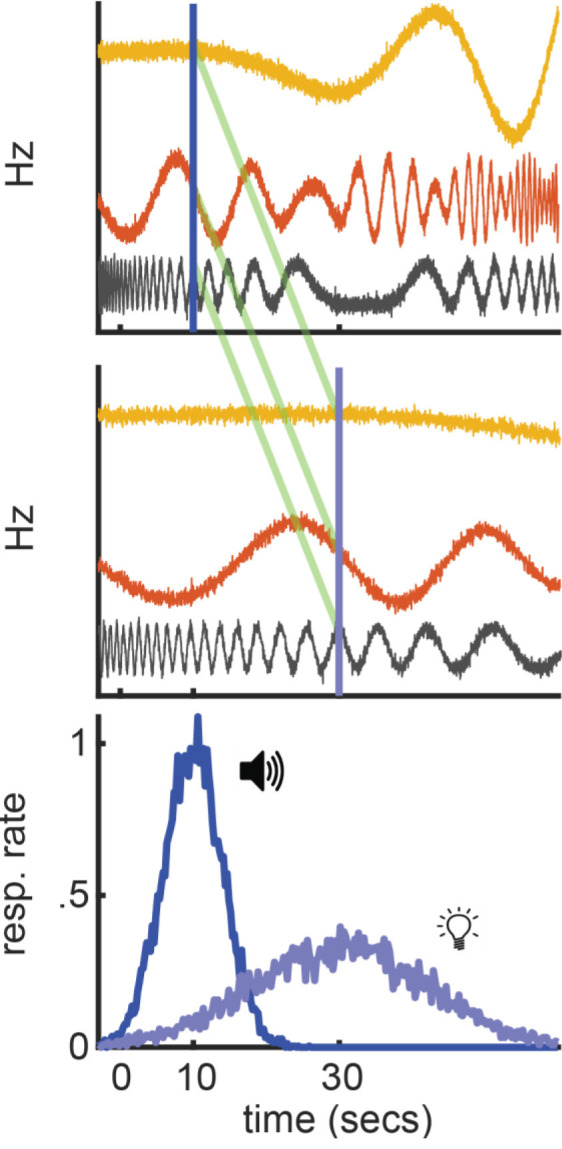
Hypothetical relative population-clock model. Top and middle: Hypothetical population activity that rescales when timing a short or long interval, respectively. Green lines highlight the same population pattern guides both intervals. Bottom: How this model would correspond to behavior during a peak-interval procedure where a tone and light are associated with 10 and 30 s, respectively.

Importantly, we should ask whether there is a way to construct a population-clock model that engages in relative-timing. Figure 7 outlines what this might look like, using the simple model discussed above. All three neurons still fluctuate erratically across time. Yet, their firing profiles now compress or stretch to match the short or long interval, respectively. In other words, much like a ramping neuron adjusting its slope, the “speed” of their firing profiles systematically scales with the timed interval. However, much like a traditional population-clock model, the pattern of activity across the population still reflects elapsed time. Importantly, if we assume that upstream circuits can set the population speed as needed for a given computation, we can apply the same interpretations as we would with a ramping model to the above behavioral effects. For example, to account for temporal averaging, we would simply say that the population moves at an intermediate speed, relative to short or long trials. Critically, empirical data are beginning to suggest that non-linear population activity indeed engages in relative-timing, suggesting this modification is biophysically realistic.

**Figure 8:**
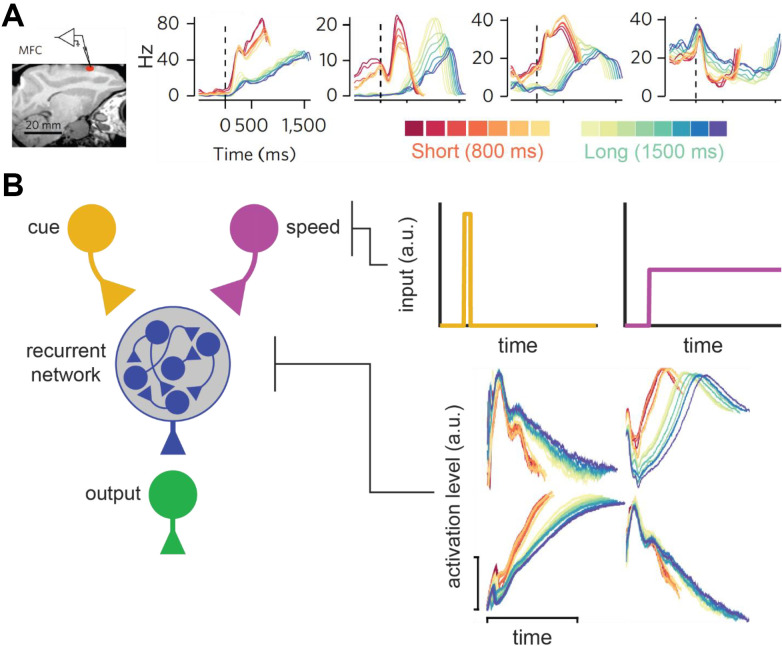
Temporal scaling in Wang et al. (2018). **(A)** Temporal scaling in neural data. Left: Electrode placement in medial-frontal cortex in Wang et al. (2018) study. Firing profiles of neurons at the recording site that displayed temporal scaling, split by both interval (short = red-hued/long = blue-hued) and within-subject variance for each interval (see color bars at the bottom). All figures adapted from Wang et al. (2018). **(B)** Wang et al.’s (2018) neural network model of temporal scaling. Left: Model-schematic. Users send a cue-pulse and tonic (i.e., speed) input to the recurrent network, which converges on the output node. Right: Profiles for the cue and tonic user-inputs (top two panels) and representative scaling activity in the recurrent layer adapted from Wang et al. (2018).

Wang et al. (2018) recently provided an excellent illustration of this principle, as summarized in Figure 8. They recorded from the medial frontal cortex, striatum, and thalamus during a task where macaques timed a short- or long-cue from trial-to-trial (0.8 and 1.5 s target intervals, respectively). Consistent with prior work, they observed ramping neurons that scaled their slopes according to the timed-interval. However, consistent with population-clock models, they also found neurons with complex, nonlinear profiles. Critically, these nonlinear neurons often scaled their profiles according to the target-interval, compressing for the shorter interval and stretching for the longer one (Figure 8A). In other words, they appeared to flexibly speed up or slow down to time different intervals, much like a ramping neuron adjusting its slope. This “temporal scaling” phenomenon primarily occurred in the MFC and striatum. Nonetheless, the thalamus appeared to set the speed of population dynamics in these areas by modulating its tonic output. Wang et al. (2018) integrated these data into a novel neural model of timing.

Like the hypothetical model described above, their theory is a hybrid between a ramping and traditional population-clock model and is summarized in Figure 8B. Specifically, complex population dynamics still represent elapsed time, and decisions are executed when a particular activity pattern emerges across neurons, like a population-clock model. In addition, consistent with ramping models, the profiles of all neurons slow down or speed up according to the target-interval. Wang et al. (2018) implemented this time-keeping population with a recurrent neural network. Paralleling the apparent role of the thalamus in their data, they delivered tonic input to the network that varied systematically with the timed-interval, along with a “cue-pulse” that initiated communication between the tonic and recurrent layer. When trained on their timing task, they found that units in the recurrent layers naturally exhibited temporal scaling, regardless of whether a given unit had developed a linear or nonlinear profile. Taken together, Wang et al.’s (2018) study provides an exhaustive empirical and theoretical illustration of temporal scaling.

However, several recent studies have documented temporal scaling; many of which predate Wang et al.’s (2018) findings. For instance, in rodents, temporal scaling occurs in a variety of areas, such as frontal association cortices (Xu et al., 2014; Li et al., 2016; Bakhurin et al., 2017; Emmons et al., 2017), the striatum (Gouvêa et al., 2015; Mello et al., 2015; Bakhurin et al., 2017), the hippocampus (Shikano et al., 2021; Shimbo et al., 2021), and even the primary visual cortex (Gavornik et al., 2009). Mello et al. (2015) provide a particularly relevant example. They recorded in the striatum of rats trained on a task that is similar to Wang et al.’s (2018), where the target-interval varied randomly across blocks of trials during a session. A subset of striatal neurons showed Gaussian-like activity profiles, with different neurons peaking at specific times between the start and end of the interval. Importantly, when the timed-interval changed, these neurons remapped in relative-time, peaking at the same *fraction* of the prior interval rather than the same absolute time within a trial. To illustrate, if the target interval switched from 12 to 48 s, a neuron that initially peaked at 6 s would switch to peaking at 24 s (i.e., half-way through either interval). Furthermore, Hardy et al. (2018) recently showed that Wang et al.’s (2018) model captures patterns of motor timing exhibited by humans that are difficult to account for with ramping models (for further discussion see Zhou and Buonomano, 2022; Zhou et al., 2022).

Taken together, these data strongly suggest that temporal scaling is a common neural phenomenon that deserves further exploration. Furthermore, by giving population clock-models a relative time-representation, these data help reconcile this model-class with the behavioral effects discussed above. Critically, if we keep the behavioral data in focus, we can derive clear future directions for neural work on temporal scaling, which we highlight next.

## What Sets The Speed? A Key Direction for Future Neural Work on Timing

Our essential point in the above section is that any model containing a relative timing signal will show promise in accounting for higher-order timing effects, regardless of whether it is a ramping or population-clock theory. Nonetheless, this argument contains a “leap-of-faith” that deserves clear emphasis. Specifically, from the behavioral inferences above, the circuits that set the speed of scaling activity should play a prominent role in higher-order timing behavior. In fact, our argument assumes that these speed-control circuits implement virtually all computations discussed above, with downstream scaling populations doing little of the “work.” Generally, prior empirical research has given more focus to characterizing scaling activity itself—be it ramping activity or nonlinear dynamics—than the upstream signals that drive it. At this point, one can posit that these circuits are as computationally sophisticated as one would like. However, to formally vet the plausibility of this assumption, we must achieve a more mechanistic understanding of speed-control. Without this, ramping and relative population-clock theories will only be able to give a *prima facie* account of higher-order timing effects, making it a key direction for future work.

**Figure 9:**
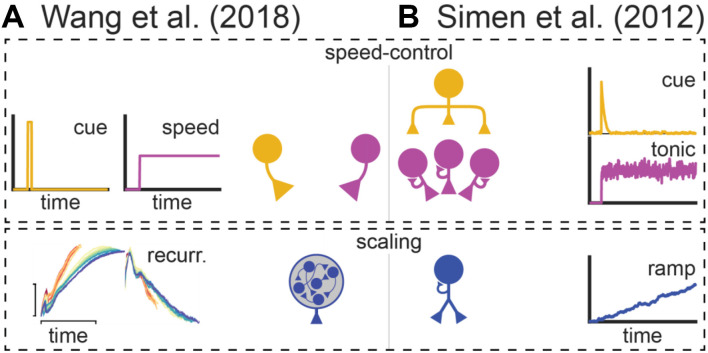
Speed control across relative population-clock and ramping models. **(A)** Wang et al. (2018) relative population-clock model as depicted in Figure 8. **(B)** Simen et al. (2011) ramping model as described in Figure 3. Boxes spanning both panels emphasize the conceptual similarities between both models with respect to the speed-control and scaling layers (top and bottom, respectively).

Interestingly, there are often strong parallels in how speed-control has been implemented within ramping and relative population-clock models. We highlight this point in Figure 9 by diagramming Wang et al.’s (2018) relative population-clock theory alongside Simen et al.’s (2011) ramping model. Conceptually, both contain a scaling layer, corresponding to the recurrent network in Wang et al.’s (2018) model and the ramping layer in Simen et al.’s (2011) theory. Furthermore, there are more apparent similarities at the upper layers, which implement speed control. For example, both contain some form of a “start-pulse” that initiates the scaling circuit. More importantly, both control the speed of the scaling circuit with a tonic signal, whose magnitude covaries with the to-be-timed interval. What both models lack is a mechanistic explanation for higher-order timing effects based on connectivity within the speed-control layers.

However, these similarities suggest that any solutions found for one model-type will likely generalize to the other. For example, to obtain temporal averaging in either model, one would want the tonic signal to output at an intermediate rate when two cues are present, causing the scaling layer to evolve at an intermediate speed during compound trials. As the experimenter sets the tonic signal manually in Wang et al.’s (2018) model, the circuit is currently not developed enough to predict this pattern. As noted above, we are exploring ways to account for this effect by modifying how the cue and tonic layers of Simen et al.’s (2011) model interact, which appear promising (De Corte, 2021). Importantly, even if successful, we would be surprised if an analogous solution could not be adapted for the speed-control layer of Wang et al.’s (2018) model. Therefore, while deterministic explanations for higher-order timing are lacking in either model, the emerging parallels allow for cross-talk between the two theoretical domains.

Notably, these similarities are not isolated to these two relative/scaling models. For example, while relative population-clock models are still fairly new, several mechanistic ramping models have been proposed. Typically, these models differ with respect to the mechanism that drives ramping activity itself. For example, Durstewitz (2003) proposed that ramping activity emerges via intracellular calcium signaling, differing from Simen et al.’s (2011) proposition that recurrent excitation drives climbing activity. In contrast, Reutimann et al. (2004) proposed that ramping activity emerges via the adaptation of inhibitory projections onto excitatory cells (i.e., a graded decrease in inhibition leads to a graded increase in excitatory activity). Critically, while these models make different assumptions about the mechanisms that drive ramping, all propose that the slope of ramping activity is driven by the magnitude of an external tonic drive.

Importantly, to empirically test and biophysically constrain any theoretical proposals, we must understand where speed-control is implemented in the brain. From the above work, we should look for a circuit that can: (1) detect when a temporal cue is initially presented, and (2) modulate downstream scaling neurons according to their interval. Of course, this process likely involves complex interactions between several areas and pathways implicated in timing (e.g., frontostriatal Matell, 2014; nigrotectal Toda et al., 2017). Nonetheless, while we can only make speculative hypotheses at this point, we view the thalamus as a good starting point for several reasons. For one, the thalamus is a known sensory relay between brain regions, making it a likely participant in the chain between cue-detection and the initiation of timing circuitry. Furthermore, Wang et al.’s (2018) data already suggest that thalamic output sets the speed of scaling activity in downstream areas. However, the thalamus is a remarkably diverse structure, composed of several subnuclei that vary markedly in function. Wang et al. (2018) primarily guided their recordings by finding thalamic neurons that projected monosynaptically to the PFC, via antidromic stimulation. Therefore, a key question is which thalamic nuclei participate in speed-control.

For our assessment, Wang et al. (2018) predominantly recorded in or near Area X, (roughly) corresponding to the rodent ventrolateral thalamus, making it an obvious candidate. However, we also view the mediodorsal nucleus (MD) of the thalamus—often sitting just above Area X in macaques—as a region of interest. Neuroanatomically, the MD integrates multisensory information, primarily trafficking between association cortices. As effects such as temporal averaging, Vierordt’s law, and covariance expectations often operate cross-modally, one might expect a multisensory area to be involved. Functionally, the MD plays a broad role in cognition (Markowitsch, 1982; Peräkylä et al., 2017), and while data are limited, manipulations of the MD disrupt baseline timing performance (Yu et al., 2010; Lusk et al., 2020; De Corte et al., 2021). As a notable example, Lusk et al. (2020) recently inhibited the MD optogenetically during the peak-procedure and found that peak times shifted later. For a relative timing model, this result is consistent with partial inhibition of a tonic drive that modulates downstream scaling activity. Importantly, the MD’s role in cognition presumably relates to the fact that it is the primary source of thalamic input to the PFC—where temporal scaling has been repeatedly documented (Ray and Price, 1993; Georgescu et al., 2020). Consistent with this, disorders that preferentially disrupt MD-PFC communication, such as Schizophrenia, also disrupt timing (Ward et al., 2012; Singh et al., 2019). Furthermore, we recently provided initial causal data suggesting that selectively blocking communication between the MD and the prelimbic cortex—a rodent analog of the PFC—markedly disrupts timing (De Corte et al., 2021). Importantly, while monosynaptic MD-PFC projections are particularly relevant to Wang et al.’s (2018) data, the MD is well positioned to modulate scaling in other areas, projecting heavily to the striatum, virtually all association cortices, and communicating with the hippocampus, presumably via reciprocal connections with parahippocampal structures (for excellent reviews see Saunders et al., 2005; Mitchell and Chakraborty, 2013; Pergola et al., 2018; Georgescu et al., 2020). Whatever nuclei might be involved, we view studying the thalamus in the context of speed-control as a promising future direction.

Beyond the thalamus, we view midbrain dopamine centers as another likely participant in speed-control. Dopamine has been implicated in a variety of timing processes, ranging from temporal memory storage/retrieval (Malapani et al., 2002), temporal prediction error-coding (Schultz, 2016; Sharpe et al., 2017), and time-based decision-making (Howard et al., 2017; Guru et al., 2020). More importantly, data have repeatedly implicated dopamine in modulating “clock-speed,” which, with respect to the current article, corresponds to biasing the speed of scaling activity. For example, during the peak-procedure, systemic injections of dopamine D2-receptor agonists or antagonists often cause peak-times to shift leftward/rightward, respectively (Meck, 1986; MacDonald and Meck, 2005; Matell et al., 2006). While understudied, recent work suggests this effect may be mediated by nigrostriatal dopamine specifically, with focal D2-antagonist infusions producing rightward shifts in the striatum (De Corte et al., 2019) and no reliable effects in frontal-cortical areas (Heslin, 2021). Interestingly, Soares et al. (2016) showed that optogenetically activating/inactivating striatal dopamine inputs produces the reverse pattern—under/overestimation of time, respectively. Nonetheless, regardless of directionality, these data collectively suggest that dopamine modulates the speed of scaling activity, making it another prime target for future work.

## Relative Vs. Absolute Timing: Do We Need Another Dichotomy?

From the above discussion, we can see that a key distinction between timing models is whether they propose an absolute or relative representation of time. For the behavioral effects discussed thus far, relative models appear more plausible than absolute ones. However, should we always expect the brain to represent time in a relative manner? After all, timing is critical in a variety of situations. Relative timing might be useful in some contexts, yet absolute timing could still be important in others. Therefore, one might expect the brain to represent both scales, either in parallel or by converting from one to the other. Consistent with this, many neural studies that find evidence of scaling activity also find a subset of neurons that encode absolute-time (MacDonald et al., 2011; Gouvêa et al., 2015; Shimbo et al., 2021). Furthermore, other facets of the behavioral literature support this proposal, cautioning us against a dichotomy.

**Figure 10:**
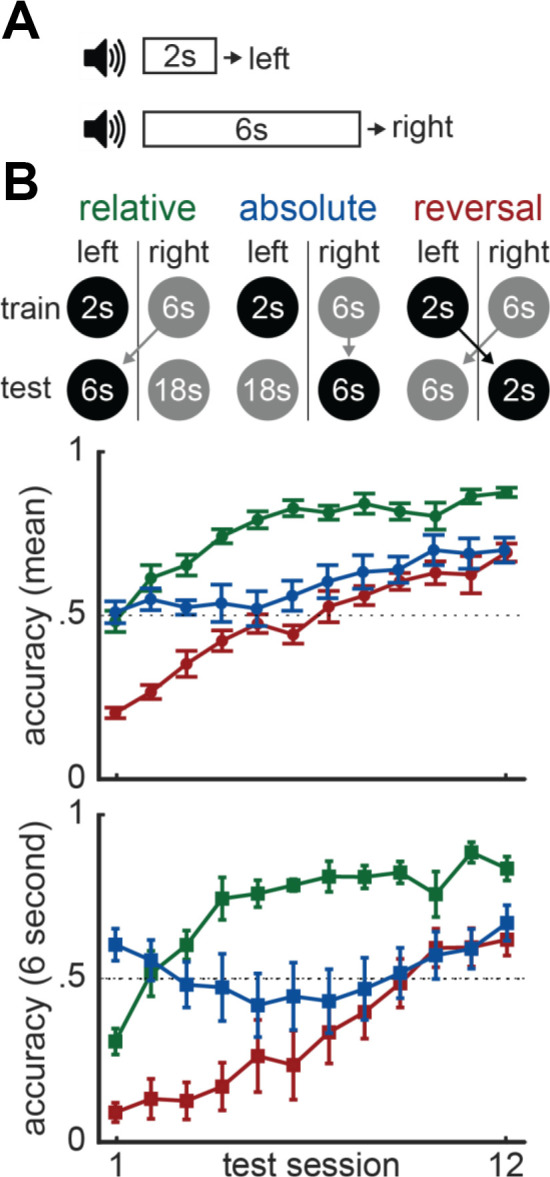
Absolute vs. relative timing. **(A)** Temporal discrimination task-design. **(B)** Task-designs for the three groups included in Akdoğan et al. (2020) at the top. Mean accuracy during phase 2 across all trial types are plotted in the middle. Accuracy for 6-s trials alone during phase 2 is plotted at the bottom.

For example, many have directly tested whether time is represented in absolute or relative terms (Fetterman et al., 1989, 1993; Zentall et al., 2004; Maia and Machado, 2009; Pinheiro de Carvalho and Machado, 2012; de Carvalho et al., 2016). These studies often use a “temporal discrimination” task. During this task, subjects are presented with a cue that lasts either a short or long duration and, once it terminates, are trained to map each duration to a distinct response-option. To use a concrete example, in Akdoğan et al. (2020), we recently trained mice to press a left lever if a cue lasted 2-s and a right lever if it lasted 6-s (Figure 10A; 2-s = left/6-s = right). Consider how two brains would solve this task–one that exclusively represents absolute time and another that represents relative time. The relative brain would use a relational decision-rule such as, “the *shorter* interval goes to the left and *longer* one goes to the right.” In contrast, the absolute brain would use a more nominal mapping such as, “the 2-s interval goes to the left and the 6-s one goes to the right.” To test these accounts, we introduced a second phase where we changed the short and/or long durations, disrupting either their relative or absolute mapping to the response-locations across different groups.

For instance, in one group, we increased the short duration to 6-s and the long duration to 18-s (i.e., 6-s = left/18-s = right). Note that, relative to the first phase, the 6-s duration switches from the right lever to the left (Figure 10B). Therefore, the absolute brain must remap this duration-response association, in addition to mapping the new 18-s duration appropriately. The relative brain would have an easier time—even though the durations have changed, the shorter interval still goes left and longer one goes right. Therefore, we refer to this as the “relative group.” In a comparison group, we continued associating the 6-s duration with the right lever during the second phase and mapped the 18-s duration to the left lever (i.e., 18-s = left/6-s = right). Here, as the 6-s interval’s mapping never changed across phases, the absolute brain only needs to map the new 18-s interval (Figure 10B). In contrast, the relative brain is at a disadvantage, as the relational mappings reversed. Consistent with a relative brain, mice in this “absolute group” performed worse than the relative group, although they did show some absolute transfer during early sessions (Figure 10B, bottom; see full manuscript for more detailed analyses/conditions).

Importantly, this result does not rule out absolute timing entirely. For example, the brain might engage in both strategies, yet be preferential to relative processing. To address this, we also incorporated a third comparison group where we simply reversed the duration-response pairings (i.e., 6-s = left/2-s = right). As both the absolute and relative mappings flipped, both brains should have been disadvantaged (Figure 10B). This “reversal group” was more impaired at the start of the transfer test than both the relative and absolute groups. Therefore, the brain indeed appears to accommodate both absolute and relative time, even if it might give more weight to relative processing. In further support of this conclusion, prior studies using similar approaches to ours often find mixed support for absolute (de Carvalho et al., 2016) and relative timing (Zentall et al., 2004).

As a more indirect approach, we can look for behavioral effects in the literature that necessarily imply that the brain processes absolute time. After all, while the higher-order effects we focus on here are more plausible under relative models, they represent a narrow subset of the behavioral literature. By expanding our horizons, we can find clear cases that imply absolute processing at some level.

We view research on “temporal maps” as a key example (Honig, 1981; Matzel et al., 1988). This literature complements work showing that animals construct “spatial maps” of the environment that they use to track the physical locations of objects (Tolman, 1948; Blaisdell et al., 2018; Widloski and Foster, 2022). The question here is whether animals do the same with time, tracking the “temporal locations” of events as they unfold during a learning episode on an underlying temporal map.

**Figure 11:**
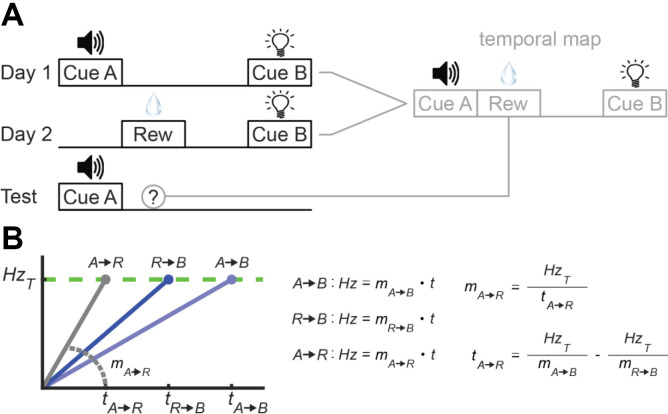
Temporal maps and absolute time. **(A)** Temporal map concept and design. Left: Task-structure across the three phases. Right: Hypothetical integration of phases 1 and 2 into a temporal map that is then used during testing. **(B)** Converting between relative and absolute time with a ramping model during a temporal map experiment.

Researchers have developed a variety of designs to address this question (Matzel et al., 1988; Arcediano and Miller, 2002; Leising et al., 2007; Molet et al., 2012; Molet and Miller, 2014). However, a simple experiment with rats would run as follows, as summarized in Figure 11A. During Phase 1, experimenters repeatedly present rats with two cues that are separated by a certain time-interval (Cue A→Cue B). On a subsequent training day (i.e., Phase 2), rats return to the chamber and are presented with a reward that is followed soon after by the second cue from Phase 1 (Reward→Cue B). Importantly, experimenters set the interval separating the reward and Cue B to where, in the context of Phase 1, the reward *should* have occurred at a certain time between the cues (Cue A→Reward→Cue B). As an example, if the Cue A→Cue B duration was 4 s and the Reward→Cue B duration was 1 s, the “inferred” delay between Cue A and reward would be 3 s. As illustrated in Figure 11A, if animals generate temporal maps of the two learning episodes and can overlay them, they should be able to estimate the Cue A→Reward interval, without directly experiencing it. To test this, experimenters present rats with Cue A during a final phase, and subjects indeed show conditioned behavior, even though it was never explicitly paired with reinforcement (e.g., Molet et al., 2012).

To our assessment, temporal map learning requires the assumption that the brain represents absolute time, at least at some level. To illustrate, consider what would have to happen for a purely relative model to account for the above experiment. In Figure 11B, we overview one possibility in which a ramping model encodes each interval strictly via slope-adaptation. The two blue lines in Figure 11B represent the “knowns” during the above experiment. Specifically, subjects experience Cue A→Cue B and Reward→Cue B pairings during the first two phases, and a ramping neuron could represent these intervals by tuning its slope appropriately. The critical component is the gray line–a ramp encoding the Cue A→Reward interval. Subjects never explicitly experience this interval. However, to account for the emergence of conditioned behavior to Cue A during the final test phase with slope-adaptation, one would assume the ramping neuron assumes this slope during testing.

To set the Cue A→Reward slope appropriately, the brain would have to extrapolate from the two known intervals. On paper, this is trivial, as outlined to the right in Figure 11B. To solve for the slope of any line, one only needs a value on the y-axis and its corresponding value on the x-axis (here, firing rate and time, respectively). With the threshold (i.e., y-value) being shared across intervals, the only “unknown” is the absolute delay between Cue A and Reward. As illustrated on the bottom right, this can be computed by rearranging the linear equations associated with the two known intervals to solve for *t*. We can leave the question of how this computation could be mechanistically implemented in neural circuits for another time. The key here is that this computation equates to converting from relative to absolute time. Moreover, to explain responding to Cue A during testing, this conversion would have to occur spontaneously, requiring the assumption that the relative brain accounts for an absolute time *a priori*. Of course, there are a variety of other theoretical interpretations that we can apply to temporal map learning (e.g., threshold-adaptation with a relative model, explicitly absolute models, etc.). However, we are unable to find a solution that accounts for these effects with purely relative processing.

Collectively, the above discussion highlights that exploring whether the brain represents absolute and/or relative time is a question that is prime for further exploration for future neural and neuro-focused behavioral studies.

## Conclusion

In conclusion, behavioral data have long suggested that the timing system has a remarkable computational capacity. Inferences from these data have proved valuable in evaluating the plausibility of ramping and population-clock models. Traditionally, ramping models appeared to have the upper-hand. However, we argue that population-clock models that capture the recent discovery of temporal scaling prove equally plausible in most cases. Moving forward, we view exploring the mechanisms of speed-control and evaluating whether neural timing is absolute and/or relative as important future directions. Of course, temporal scaling prompts several other avenues for future research, such as whether ramping neurons have a special status compared to nonlinear ones. Yet, with respect to the integration of behavioral and neural timing data, we view these questions as key.

## Data Availability Statement

The original contributions presented in the study are included in the article, further inquiries can be directed to the corresponding author.

## Author Contributions

BC: concept development, writing manuscript, figure generation. BA and PB: conceptual development, manuscript revisions, and feedback. All authors contributed to the article and approved the submitted version.
